# Gastrointestinal complaints in shift-working and day-working nurses in Iran

**DOI:** 10.1186/1740-3391-8-9

**Published:** 2010-10-07

**Authors:** Hamid Reza Saberi, Ali Reza Moravveji

**Affiliations:** 1 Department of Occupational Health, Kashan University of Medical Sciences, Kashan, Iran; 2 Department of Community Medicine, Kashan University of Medical Sciences, Kashan, Iran

## Abstract

**Background:**

There is evidence in the scientific literature of the adverse physiological and psychological effects of shift work. The work of nurses in hospitals is connected with shift and night work. Several publications have described gastrointestinal disturbances in shift workers. The aim of this study was to compare the frequency of gastrointestinal (GI) complaints of nurses on a rotating shift with that of nurses on a regular day shift.

**Methods:**

The study involved 160 nurses (133 working in shifts and at night and 27 working on day shifts) in the Shahid Beheshti Hospital in Kashan, Iran. These nurses answered a Gastrointestinal Symptom Questionnaire regarding the presence of gastrointestinal symptoms (including heartburn, regurgitation, constipation, diarrhea and bloating). Positive responses required frequent symptom occurrence in the past 4 weeks. Significance of group differences was assessed by chi-square and Fisher-exact tests.

**Results:**

Prevalence of GI symptoms was significantly higher (p = 0.009) in rotating-shift nurses (81.9%) than in day-shift nurses (59.2%). Irregular meal consumption (p = 0.01) and GI medications (p = 0.002) were all significantly higher among the rotating shift nurses. In both groups, regurgitation was the most common symptom.

**Conclusion:**

Nurses on rotating shifts in Iran experience more GI disturbances than do nurses on day shifts.

## Background

There is increasing evidence that circadian rhythm disturbance can cause a variety of health disorders [1,2]. Nurses, because of their profession, have to perform their important and difficult tasks at any time in the 24 hours of the day. Erratic working shifts can cause decreased proficiency, somatic and psychological disorders and increase in nursing and medical error [3,4].

Previous studies have addressed a variety of maladies associated with shift work, including gastrointestinal (GI) symptoms [5,6]. For example, a study showed that working in different shifts can harm the GI normal movements and cause disorders in excreting digestive enzymes and acid-alkaline balance [7]. These alterations may be caused by sleep disorders, as they have a negative correlation with last night's sleep quality in these subjects [8]. Furthermore, in an investigation designed by German researchers, peptic ulcer incidence was higher in shift-workers and night-workers [9]. In a study in Japan, conducted by endoscopy, peptic ulcer prevalence was higher in shift-workers (2.38%) than in day-workers (1.03%). Duodenal ulcer also had greater prevalence (1.37% and 0.69%, respectively) [10]. A study in Iceland showed that nurses working 16 hours in a morning-evening shift had more severe GI symptoms, possibly because of a lack of enough resting time between the end of the evening shift and the start of the morning one [11]. Another study identified correlations between GI symptoms and psychological disorders such as anxiety and depression [12].

To supplement the existing literature, we investigated GI symptoms in shift-work and day-shift nurses in a training hospital in Iran.

## Methods

### Subjects

In this cross-sectional study, all male and female nurses with different working shifts working at the Shahid Beheshti Hospital in the city of Kashan from March 2008 until March 2009 were included. Known cases of GI diseases or any other kind of disorders that may interfere or mimic GI symptoms, for example respiratory tract disorders, and known cases of any kind of heart disease, hepatitis, etc. were excluded from our study. From all 200 nurses in the hospital, 160 were included. The patients were moderately matched by age and gender.

### Data collection and questionnaire

The data were collected by the means of a questionnaire addressing demographic information and GI symptom complaints extracted from a questionnaire designed by Bovenschen et al. [13]. The questionnaire contains 16 prevalent GI symptoms, and the severity of each symptom experienced during the previous 4 weeks was measured by a 7-point Likert scale (0-6). To shorten the time for filling the forms, we included only the 5 most common GI symptoms, namely, diarrhea and constipation, bloating, belching, heartburning, and epigastria pain and regurgitation, scaling from "never" to "most of the time". Any subject with at least one complaint as "most of the time" in the past 4 weeks was considered as a patient with positive history of GI symptoms. Answers to other questions such as tea and coffee consumption and marriage status were also collected.

### Statistics

All statistical analyses were performed using the SPSS software package (version 16.0 for Windows, SPSS, Chicago, IL, USA). χ^2 ^and Fisher exact tests were performed to study differences. P value of less than 0.05 was considered statistically significant.

## Results

### Basic characteristics

Of the 160 nurses included in our study, 43 (26.8%) were male. Only 27 (16.8%) subjects were only-morning workers and 133 (83.1%) had more than one working shift. Also 64 (40%) had erratic work shifts. The mean age of morning workers and shift workers were 35 and 38.5 years, respectively. Twenty-one morning shift workers and 108 double shift workers were married. Regular daily tea consumption was 97.7% in shift-workers and 10% in morning workers and regular daily coffee consumption was 12% in shift-workers and 37% in morning-workers.

### Complaints

Prevalence of complaint of at least one GI symptom was 81.9% in shift-workers and 52.2% in day-shift workers, this difference being significant (p = 0.009). In female subjects these proportions were 84.8% and 55.5%, and in male nurses they were 72.5% and 66.7%, respectively. In non-married nurses, GI symptoms were present in 68% of shift-workers and 50% of day-shift workers. In married nurses, these proportions were 85.2% and 61.9%, respectively.

Table 1 presents the distribution of gastrointestinal symptoms in shift-workers and day-shift workers. It shows that the most frequent complaint was regurgitation (52%) and the least frequent was melena (2%). Prevalence of none of the individual symptoms was significantly different between the two groups. There was also no significant difference in GI symptoms between male and female subjects (p = 0.14).

**Table 1:** Distribution of gastrointestinal symptoms^a^

*Complaints*		*Day Worker*	*Shift Workers*	*Total*	*P*V
*Diarrhea*	*positive*	3(11)	28(21)	31(19)	0.2
	*negative*	24(89)	105(79)	129(81)	
*Constipation*	*positive*	10(37)	50(37)	60(37)	0.9
	*negative*	17(63)	83(63)	100(63)	
*Bloating*	*positive*	9(33)	70(52)	79(49)	0.06
	*negative*	18(67)	63(48)	81(51)	
*Regurgitation*	*positive*	13(48)	71(53)	84(52)	0.6
	*negative*	14(52)	62(47)	76(48)	
*Heart-Burn*	*positive*	8(29)	55(41)	63(39)	0.25
	*negative*	19(71)	78(59)	97(61)	
*Melena*	*positive*	0(0)	3(2)	3(2)	1
	*negative*	17(100)	130(98)	157(98)	

^a ^Entries are number of workers. Percentage of total workers is indicated in parenthesis. P-values was calculated by χ^2 ^and Fisher exact test, as appropriate

Table 2 shows the number of GI symptom complaints. As seen, this number is significantly higher in shift-workers. Table 3 shows subjects with at least one "most of the time" GI symptoms divided by age groups. Shift workers had significantly more complaints than day workers (p = 0.015).

**Table 2:** Number of GI symptom complaints^a^

*Number of Complaints*	*1*	*2*	*3*	*4*	*5*	*6*
*Morning workers*	5(17.2)	2(10.5)	3(12)	4(11.4)	1(10)	1(14.2)
*Shift workers*	24(82.7)	17(89.5)	22(88)	31(88.6)	9(90)	8(85.8)
*Total*	29	19	25	35	10	7

^a ^Entries are number of workers. Percentage of total workers is indicated in parenthesis.

**Table 3:** Subjects with at least one GI symptom, divided by age groups^a^

*Age Groups*		*Morning only*	*Shift working*	*Total*
*20-29*	*positive*	1	38	39
	*negative*	2	2	10
*30-39*	*positive*	13	58	71
	*negative*	8	8	16
*40-49*	*positive*	1	13	14
	*negative*	1	8	9
*50<*	*positive*	1	0	1
	*negative*	0	0	0

^a ^p = 0.015, tested by Fisher exact test.

### Medications

Prevalence of consumption of gastrointestinal medication in male subjects was 58.8% and 33.3% in shift-workers and day-shift workers, respectively. These proportions were 62.6% and 27.8% in female subjects. Non-steroidal anti- inflammatory drugs (NSAIDs) and antacids were the most frequent medication used by all groups (Tables 4 and 5).

**Table 4:** Number of subjects using at least one gastrointestinal medication^a^

*Gender*		*yes*	*no*	*total*
	*Day*	3	6	9
*Male*	*Shift*	20	14	34
	*Total*	23	20	43
	*Day*	5	13	18
*Female*	*Shift*	62	62	99
	*Total*	67	50	117

^a ^There is a significant difference between male and female (p = 0.032) and between day workers and shift workers (p = 0.002).

**Table 5:** Distribution of gastrointestinal medication types used by nurses

*Type of medication*	*Day workers*	*Shift workers*	*Total*
*NSAID*	4	50	54
*H2 Blockers^1^*	3	24	27
*Antacis^2^*	2	32	34
*Cliniduim-C*	1	17	18
*Metoclopramide*	0	10	10
*Omeprazole*	1	12	13
*Laxatives^3^*	3	12	15
*Dimethicone*	0	3	3

^1 ^histamine type 2 receptor antagonists: ranitidine, famotidin nizatidine, etc.

^2 ^all aluminum/magnesium hydrocholoride, etc.

^3 ^all kinds of laxative from various categories such as docustate, bisacodyl, sennacastor oil, etc.

## Discussion

Worldwide, gastrointestinal disorders are common complaints in the general population and must be monitored by community health care organizations [14,15]. For this purpose, questionnaires can be very helpful because they are easy to use extensively, can gather a lot of information rapidly, and are relatively inexpensive (particularly in comparison to monthly check ups) [16-18].

In the present study, gastrointestinal complaints were reported by a very high proportion (81.9%) of shift-workers. This proportion was twice as large as that reported in a previous study in Korea [19]. This could be due to environmental factors, hospital organization, social factors, insufficient welfare facilities, inordinate hours of working or erratic shifts, or even perhaps inappropriate answers to various questionnaires. On the other hand, Scott and colleagues reported 75% of gastrointestinal complaints in night-workers [20].

In our population of nurses in Iran, the prevalence of GI symptoms was significantly higher in shift-work nurses than in day-shift nurses, which is consistent with findings in other geographical locations [11,12]. The causes of this difference, however, are not evident. Factors such as sleep disorders [21-23], inappropriate nutrition or irregularity in the timing of meals [24], and mental and psychological disorders [12] might be responsible for the higher incidence of GI symptoms.

Irregularity in the timing of meals was previously described and was attributed to lack of time, appetite disturbance and work stress [5,24]. This could cause the nurses to have more GI symptoms, but, surprisingly, in our study nurses with meal timing irregularity had fewer symptoms (p < 0.001).

We found that use of gastrointestinal medication was much more common among shift-work nurses than among day-shift nurses (p = 0.002; Table 4). One might speculate that shift workers have more problems than others and try to eliminate them by using drugs. However, quite a few shift workers in our population used NSAIDs. These medications are mainly used for musculoskeletal pain and can cause or worsen GI symptoms. Thus, perhaps greater use of NDAIDs may be responsible for the greater incidence of GI symptoms in shift-work nurses.

It was somewhat surprising that no particular symptom was more prevalent in shift-work nurses than in day-shift nurses (Table 1). However, in Bilski's study, defecation irregularity was the only complaint more prevalent in shift-work nurses, and the frequency of other complaints, such as diarrhea, non-specific pains and gastric ulcers, did not differ from that in day-shift nurses [24].

Gastrointestinal symptoms were higher in nurses under 40 years of age (p = 0.015). This age effect was also reported by Zhen Lu and colleagues [12]. One would expect aging to make people more prone to gastrointestinal disorders, but it was the younger group who reported more GI symptoms in our study. Perhaps younger nurses are more likely to volunteer to work above average working hours or on irregular shifts.

We have to note that our study has some limitations. We had difficulties finding nurses that only worked in morning shifts. As mentioned earlier, only 27 (16.8%) nurses had morning-only shifts. Our data were obtained from a single hospital, and we are considering collecting data from more hospitals nationwide in the future. Our study was cross-sectional and thus, necessarily, only descriptive. Prospective longitudinal studies must be carried out to evaluate the causes and long-term effects of GI symptoms in shift-work nurses.

## Competing interests

The authors declare that they have no competing interests.

## Authors' contributions

HRS and ARM participated in design, acquisition of data, analysis and interpretation of data, and manuscript preparation. Both authors read and approved the final manuscript.
